# Reduced expression of pyruvate kinase in kidney proximal tubule cells is a potential mechanism of pravastatin altered glucose metabolism

**DOI:** 10.1038/s41598-019-39461-2

**Published:** 2019-03-29

**Authors:** Yong Pyo Lee, Yuri Cho, Eun Jee Kim, Hyojung Lee, Hoon Young Choi, Hye Jin Wang, Eun Seok Kang, Yu Seun Kim, Myoung Soo Kim, Beom Seok Kim

**Affiliations:** 1 Department of Medicine, The Graduate School, Yonsei University, Seoul, Republic of Korea; 2 The Research Institute for Transplantation, Yonsei University College of Medicine, Seoul, Republic of Korea; 3 Brain Korea 21 PLUS Project for Medical Science, Yonsei University, Seoul, Republic of Korea; 4 Department of Internal Medicine, Yonsei University College of Medicine, Seoul, Republic of Korea; 5 Division of Endocrinology and Metabolism, Department of Internal Medicine, Yonsei University College of Medicine, Seoul, Republic of Korea; 6 Department of Transplantation Surgery, Severance Hospital, Yonsei University Health System, Seoul, Republic of Korea; 7 Division of Nephrology, Department of Internal Medicine, Severance Hospital, Yonsei University Health System, Seoul, Republic of Korea

## Abstract

Recent studies have reported that statins are associated with increased incidence of diabetes. Although several mechanisms have been proposed, the role of the kidney’s glucose metabolism upon statin treatment is still unclear. Thus, we investigated the role of pravastatin in gluconeogenesis and glycolysis. HK-2 and HepG2 cells were treated with pravastatin and cultured under either high- or normal-cholesterol conditions. In HK-2 cells treated with pravastatin under both high- and normal-cholesterol conditions, the protein expression of only pyruvate kinase isozymes L/R (PKLR) decreased in a dose-dependent manner, while the protein expression of other glucose metabolism related enzymes remained unchanged. Within the *in vivo* experiment, male C57BL/6 mice were fed either pravastatin-treated normal-fat diets for 2 or 4 weeks or pravastatin-treated high-fat diets for 16 weeks. Protein expression of PKLR in the kidneys from mice that consumed pravastatin-treated high-fat diets decreased significantly compared to the controls. Upon the treatments of pravastatin, only the PKLR expression decreased in lean mice. Furthermore, PKLR activity decreased significantly in the kidney after pravastatin treatments. However, there was no change in enzyme activity in the liver, suggesting that pravastatin decreased PKLR activity only in the kidney. This change may be associated with the hyperglycemic effect of statins.

## Introduction

Studies have shown that lowering LDL cholesterol concentrations with statins has a significant effect on reducing the risk of cardiovascular and cerebrovascular diseases in both diabetic and nondiabetic populations^1,2^. Statin therapy has also been demonstrated to improve endothelial function, inhibit proliferation of smooth muscle cells, and reduce oxidative stress and inflammation^3^. However, there are concerns regarding the unanticipated responses and adverse effects associated with the increased clinical use of statins. While there are reports that statins contribute to the prevention of diabetes due to their pleiotropic effect and ability to lower lipids^2,4^, other studies have suggested that statins induce the onset of muscle-related diseases, diabetes, and diseases of the central nervous system, in addition to reducing kidney function^5–10^. In particular, numerous studies have demonstrated that statin therapy is linked to the development of type 2 diabetes mellitus (T2DM)^11–14^. A meta-analysis of major statin trials with 91,140 nondiabetic participants showed that statin therapy was associated with a 9% increased risk for incident T2DM^15^. Carter *et al*. reported that treatment with higher atorvastatin, simvastatin, or rosuvastatin doses on the patients aged 66 or older without diabetes was associated with an increased risk for new-onset DM (22, 10, and 18%, respectively)^16^.

In our previous studies, we reported that statins increase hepatic glucose production by increasing levels of important glucose-producing enzymes, such as phosphoenolpyruvate carboxykinase (PEPCK), and that chronic statin therapy contributes to the development of T2DM in mice^17^. While glucose metabolism is mainly regulated in the liver, human kidneys contribute to glucose homeostasis through gluconeogenesis, glucose filtration, glucose reabsorption, and glucose uptake, ultimately accounting for up to 20% of all glucose production^18^. However, our knowledge of renal gluconeogenesis is limited, and to the best of our knowledge there are no studies reporting the effect of statins on glucose metabolism in the kidney. Thus, we investigated the role of statins in gluconeogenesis and glycolysis *in vitro* as well as *in vivo*.

## Results

### Effects of pravastatin on glucose metabolism related enzymes in HK-2 and HepG2 cells under high-cholesterol conditions

**Figure 1:**
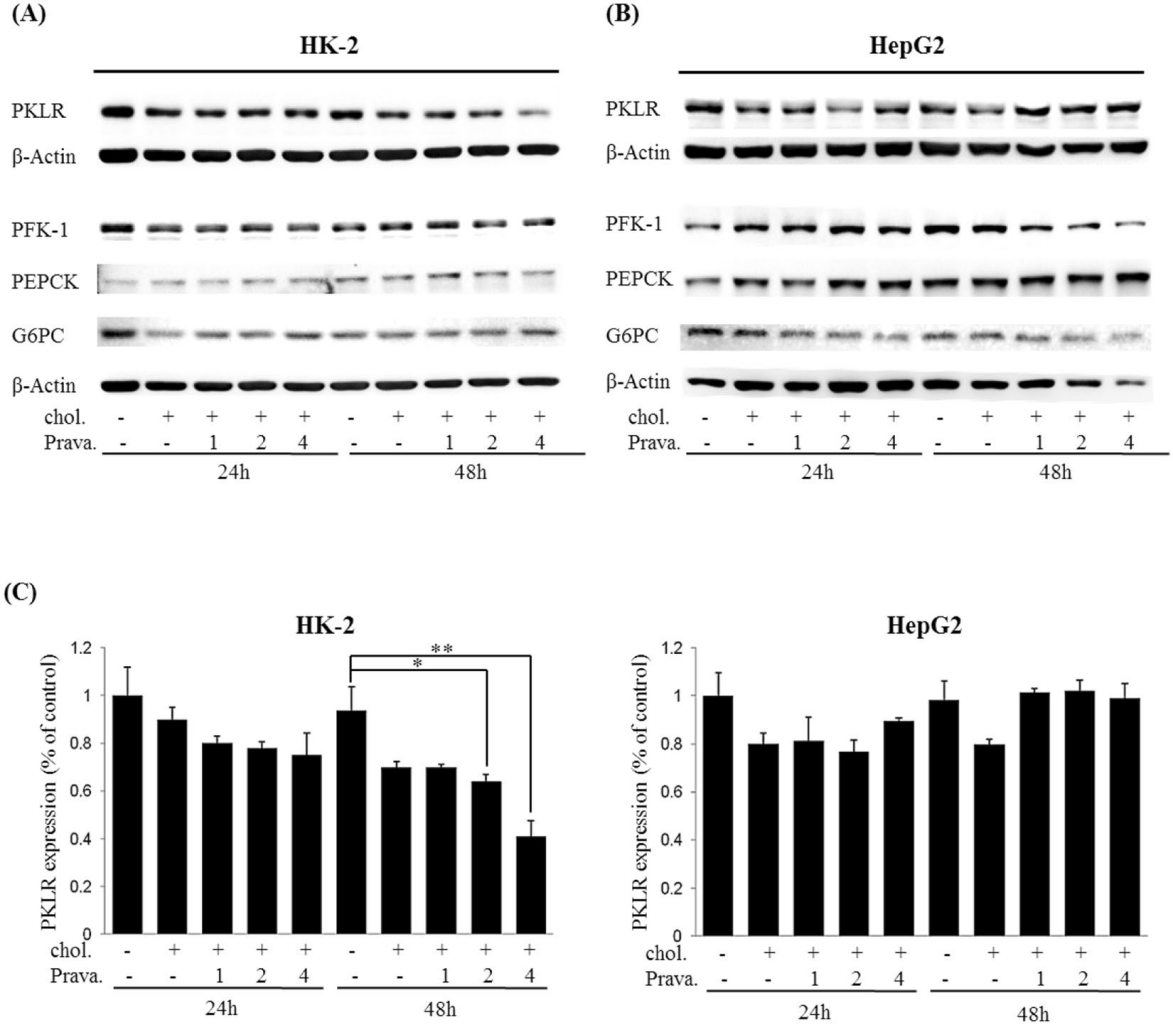
Effects of pravastatin on glucose metabolism-related enzyme expression in HK-2 cells and HepG2 cells under high-cholesterol conditions. HK-2 cell (**A**) and HepG2 cell (**B**) were treated with 1, 2, or 4 μM pravastatin (prava) plus 25-hydroxy cholesterol (chol.) for 24 or 48 h. Total protein lysates were western blotted using the antibodies; PKLR (59 kD), PFK-1 (85 kD), PEPCK (62 kD), and G6PC (36 kD). β-actin was used to confirm equal loading. (**C**) Bar graphs representing quantitative differences in expressions of PKLR. Results are means ± SEMs (n = 3). *P < 0.05, **P < 0.01 vs. the 48 h-untreated control (−/−, CTRL).

To understand the effect of pravastatin on enzymes involved in glucose metabolism in kidney proximal tubule cells and in hepatocytes under high-cholesterol condition, both HK-2 and HepG2 cells were treated with pravastatin plus 25-hydroxy cholesterol and cholesterol. As shown in Fig. 1, HK-2 cells showed no dose- or time-dependent changes in the levels of 1-phosphofructokinase (PFK-1), PEPCK, and glucose 6 phosphatase (G6PC); however, PKLR levels decreased in a dose- and time-dependent manner 48 h after pravastatin treatment. For PKLR, while HepG2 cells exhibited no change, HK-2 cells exhibited significant reductions of 0.65 ± 0.03% and 0.41 ± 0.07% after treatment with 2 and 4 μM pravastatin at 48 h, respectively, when compared with the untreated control (chol-/prava-) (Fig. 1C). The image of the full-length blots and the quantitative densitometry bar graphs for PFK-1, PEPCK, and G6PC are in Supplementary Figs 1 and 2, respectively.

### Effects of pravastatin on glucose metabolism related enzymes in HK-2 and HepG2 cells under normal conditions

**Figure 2:**
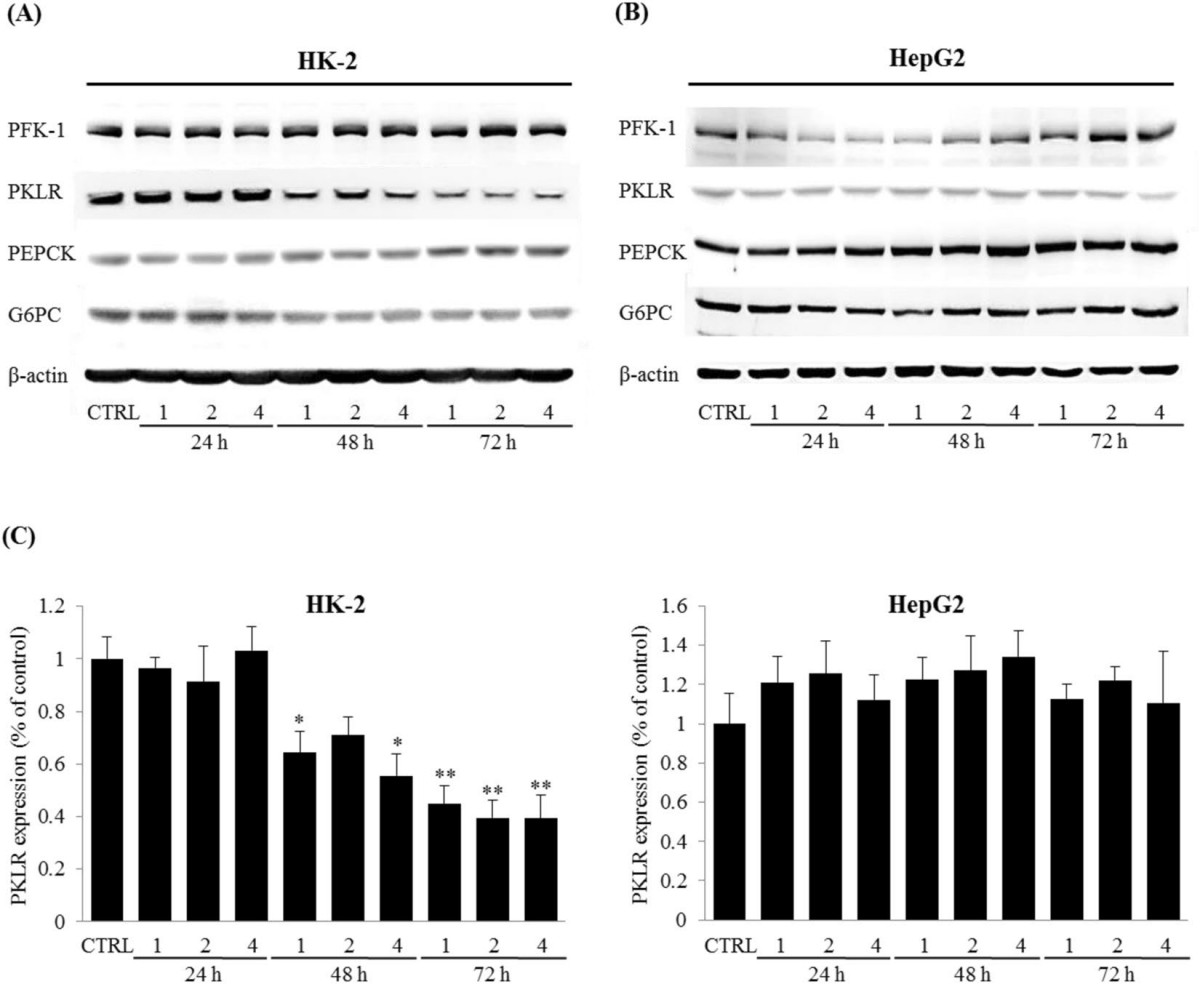
Effects of pravastatin on glucose metabolism-related enzymes in HK-2 cells and HepG2 cells under normal conditions. HK-2 cell (**A**) and HepG2 cell (**B**) were treated with 1, 2, or 4 μM pravastatin for 24, 48, or 72 h. Western blotting was used to evaluate the protein expression of PFK-1, PKLR, PEPCK, and G6PC in the treated cells. Cell lysates (40 μg) were loaded onto gels and immunoblotted; β-actin was used to confirm equal loading. (**C**) Bar graphs representing quantitative differences in expressions of PKLR. Results are means ± SEMs (n = 3). *P < 0.05, **P < 0.01 vs. CTRL.

To test the effect of pravastatin without cholesterol on the kidney and the liver, both HK-2 and HepG2 cells were treated with 1, 2, or 4 µM pravastatin for 24, 48, and 72 h, and then the protein expressions of PKLR, PFK-1, PEPCK, and G6PC were investigated. The protein expression of PKLR in HK-2 cells significantly decreased, while those of other enzymes remained unchanged (Fig. 2C left and Supplementary Fig. 4A). In HepG2 cells, pravastatin did not induce significant changes in the protein expression of PKLR, PFK-1, PEPCK, and G6PC (Fig. 2C right and Supplementary Fig. 4B). For PKLR, while HepG2 cells exhibited no change, HK-2 cells exhibited significant reductions of 0.45 ± 0.07%, 0.40 ± 0.07%, and 0.39 ± 0.09% after treatment with 1, 2 and 4 μM pravastatin at 72 h, respectively, when compared with the untreated control (CTRL) (Fig. 2C). The image of the full-length blots and the quantitative densitometry bar graphs for PFK-1, PEPCK, and G6PC are in Supplementary Figs 3 and 4, respectively.

### Effects of pravastatin on PKLR expression in high-fat diet-fed C57BL/6 mice

**Figure 3:**
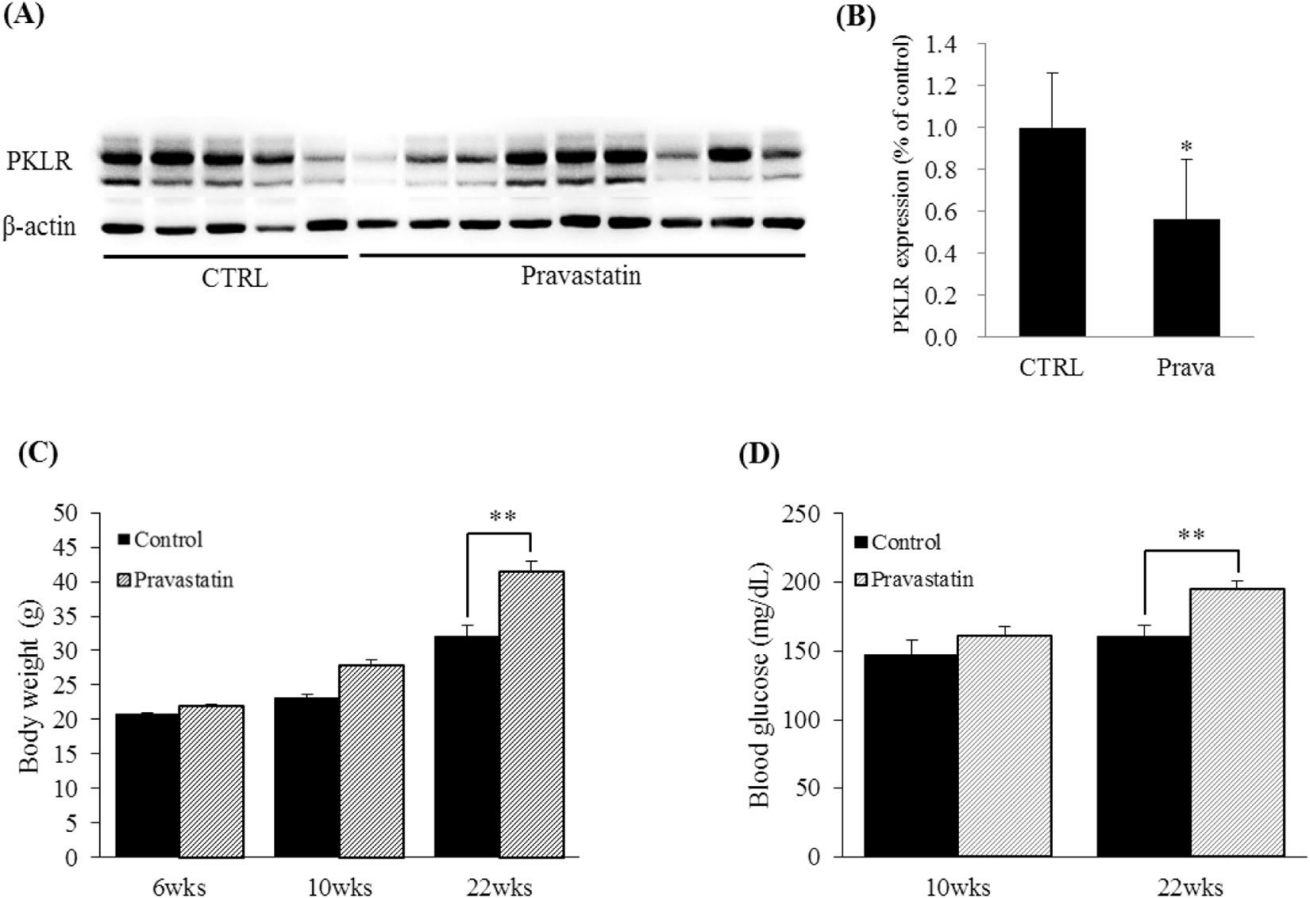
Effects of pravastatin on PKLR expression in high-fat diet-fed C57BL/6 mice. C57BL/6 mice were fed a high-fat diet with or without pravastatin (0.01%, w/w) for 16 weeks. (**A**) Kidney tissue lysates (40 μg) were loaded onto gels and immunoblotted; β-actin was used to confirm equal loading. (**B**) Bar graphs representing quantitative differences in PKLR protein expression. Body weights (**C**) and blood glucose levels (**D**) were measured at 6, 10, and 22 weeks of age and 10 and 22 weeks of age, respectively. *P < 0.05; **P < 0.01 compared with untreated mice. Data are presented as mean ± SEM (CTRL; n = 5, Pravastatin; n = 9).

The overall protein expression level of PKLR was significantly decreased by pravastatin treatment in the kidney; protein expression of PKLR decreased to 0.56 ± 0.28% when compared with that of the control (Fig. 3B). These results are in agreement with those obtained using the HK2 cell line and show that pravastatin reduced PKLR expression in kidney tubular cells under high-cholesterol or high-fat conditions. The image of the full-length blots is in Supplementary Fig. 5. Mean body weight gain was significantly higher in pravastatin-treated mice (Fig. 3C) and blood glucose levels were significantly elevated in pravastatin-treated mice at 22 weeks (Fig. 3D) compared with untreated control mice.

### Effects of pravastatin on glucose metabolism-related enzymes in lean C57BL/6 mice

**Figure 4:**
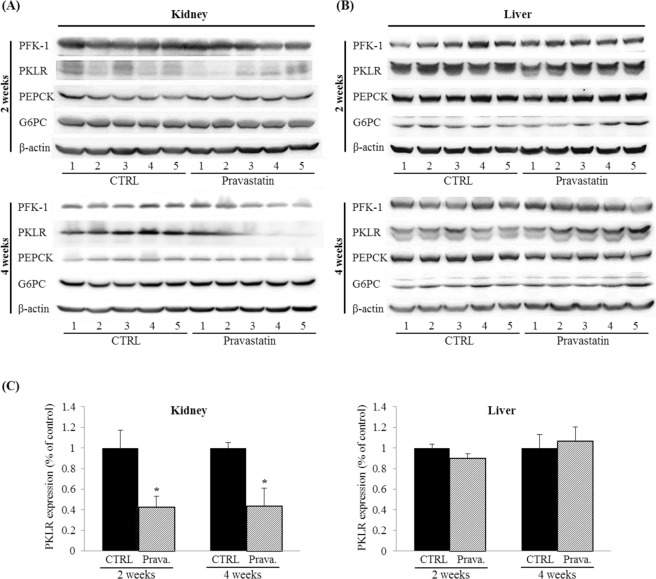
Effects of pravastatin on glucose metabolism-related enzymes in lean C57BL/6 mice. C57BL/6 mice were fed a diet containing pravastatin (0.01%, w/w) for 2 or 4 weeks (n = 5). Mouse kidney (**A**) and liver (**B**) tissue lysates (40 μg) were loaded onto gels and western blotting was used to evaluate protein expression of PFK-1, PKLR, PEPCK, and G6PC in the mouse kidney and liver. β-actin was used to confirm equal loading. (**C**) Bar graphs representing quantitative differences in expressions of PKLR in kidney and liver. The expressions of PKLR considerably deceased after pravastatin treatment for 2 and 4 weeks. Results are means ± SEMs (n = 5). *P < 0.05 vs. CTRL.

To investigate the effect of pravastatin on enzymes involved in glucose metabolism in the kidney and the liver, 8-week-old C57BL/6 mice were reared for with a formula feed containing pravastatin. There was no difference in the change in body weight between the experimental groups during the experiment periods. Per the data from the cell lines, the protein expression levels of only PKLR decreased significantly after pravastatin treatment for 2 and 4 weeks (0.42 ± 0.11% and 0.43 ± 0.18% of untreated controls, respectively in Fig. 4C left), while there was no detectable change in those of other proteins in the kidney (Supplementary Fig. 9A). No changes were observed in the expression of all four enzymes in the liver (Fig. 4C right and Supplementary Fig. 9B). The images of the full-length blots and the quantitative densitometry bar graphs for PFK-1, PEPCK, and G6PC are in Supplementary Figs 7–9, respectively.

### Alteration of PKLR activity and blood glucose level by pravastatin in lean C57BL/6 mice

**Figure 5:**
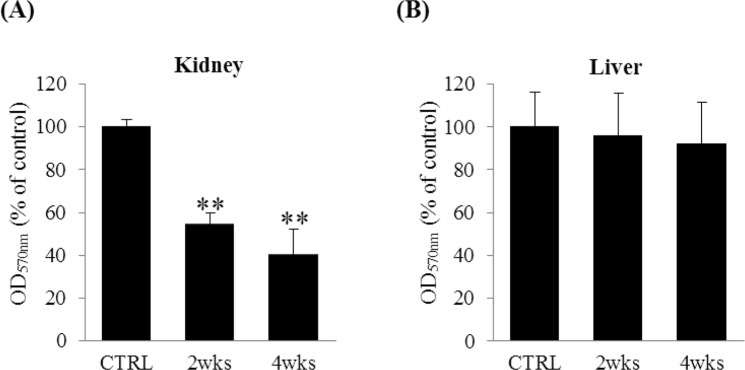
Alteration of PKLR activity by pravastatin treatment in lean C57BL/6 mouse kidney and liver. Mouse kidney (**A**) and liver (**B**) were lysed by activity assay buffer and then pyruvate measured in 50 μg tissue. The generated pyruvate is oxidized by pyruvate oxidase, while producing light at 570 nm wavelength. **P < 0.01 vs. CTRL (n = 5).

**Figure 6:**
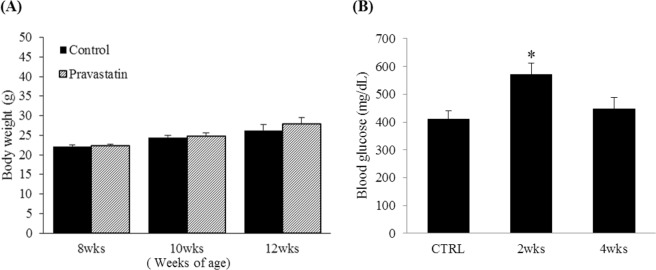
Effects of pravastatin on body weights and blood glucose levels in lean C57BL/6 mice. Body weights (**A**) and blood glucose levels (**B**) were measured after 2 and 4 weeks of feeding with or without 0.01% pravastatin. Data are expressed as percent of the control. *P < 0.05 vs. CTRL (n = 5).

Pravastatin was found to reduce the expression of PKLR. To understand the actual effect of pravastatin on PKLR activity, a PKLR activity assay was performed. Pravastatin decreased PKLR activity in the kidney to 50.1 ± 6.5% and 38.2 ± 10.1% after 2 and 4 weeks of treatment, respectively, compared to the control (Fig. 5A). In contrast, no change was observed in the liver (Fig. 5B). Body weights were measured at three different time points during the four weeks and there was no significant change in body weights (Fig. 6A). Blood was collected at the time of sacrifice and blood glucose levels were measured. Although blood glucose levels of pravastatin-treated group for 4 weeks did not significantly change, those of pravastatin-treated group for 2 weeks significantly increased by 39.0 ± 6.4% (*P < 0.05) when compared with the control (Fig. 6B).

## Discussion

Due to the widespread use of statins, there is a newfound need for further studies into the clinically associated adverse effects of statins. Although several clinical and epidemiological studies have shown that statin therapy increases the risk of T2DM, there is a lack of research on its underlying mechanism. Moreover, the effect of statins on the kidney in correlation to diabetes remains unclear. Therefore, in this study, we investigated the role of statins in renal glucose metabolism, and obtained the results that pravastatin significantly decreased PKLR expression among enzymes involved in glucose metabolism in C57BL/6 mouse kidneys and HK-2 cells.

Since it has been reported by a few research groups that PKLR affects the onset of diabetes, our study focused on the more specific role of pravastatin in regulating the protein expression of PKLR. Pyruvate kinases are important glycosylated enzymes and are expressed in major organs including liver, pancreas, and kidney. The expression of PKLR is upregulated by glucose through the carbohydrate response element in the gene promoter^19^. Also, the variants in the PKLR gene has been shown in associated with an increased risk of diabetes^20^. Hence, we believe that pravastatin strongly interferes with the production of PKLR, which may ultimately result in the eventual onset of diabetes.

In our previous study, the expressions of PEPCK and G6PC were increased due to the effect of statins, which might induce gluconeogenesis in the liver^17^. Moreover in current study, the expression of glycolysis-related enzyme, PKLR, was significantly decreased in the kidney, while there is no significant change in the liver. Based on the results of the previous and the current studies, statins increase gluconeogenesis in the liver and decrease glycolysis in the kidney, which might lead to an accumulation of glucose in the system, resulting in a diabetogenic environment.

The culture media for HK-2 cells used in this study contain 17.5 mM of glucose, which is higher than normal glucose media. It is possible that the expression of intracellular glucose metabolite molecules is affected by higher glucose level in the media. However, considering that the *in vivo* experiment results were consistent with the *in vitro* and that many of the HK-2 cell studies use the same DMEM/F12 media^21,22^, it is less likely that the glucose concentration of DMEM/F12 media had a significant impact on the *in vitro* results. Further studies might be still necessary to confirm the results with the media at a normal glucose concentration.

In conclusion, our results suggest that statins are implicated in glucose metabolism in a variety of ways that either affect the occurrence of T2DM or exacerbate clinical symptoms. Moreover, pravastatin induced PKLR reduction in kidney tubule cells, which might partly contribute to statin-induced diabetogenicity. We believe that this is the first study that aims to show how statins can affect renal glucose metabolism enzymes and that further studies should be conducted in order to more definitively identify the underlying mechanism of statin-induced diabetes in the kidney.

## Materials and Methods

### Ethics statement

This work was performed in accordance with the Laboratory Animals Manual and the Laboratory Animal Care and Use Committee, edited by the National Research Council of the National Animal Society. All animal studies were conducted using a protocol approved by the committee for the care and use of laboratory animals of Yonsei University College of Medicine.

### Cell culture

Human renal proximal tubular epithelial cell line (HK-2), which are immortalized human renal proximal tubular epithelial cell, and the hepatocellular carcinoma HepG2 cell line were obtained from ATCC (Rockville, MD). HK-2 cells at passages 10–15 and HepG2 were cultured. The cell lines were cultured in Dulbecco’s modified Eagle’s medium/F12 (1:1) (Gibco, Grand Island, NY, USA) culture medium containing 10% fetal bovine serum (Gibco), 100 U/ml penicillin, and 100 mg/ml streptomycin (Gibco). Cells were treated with 1, 2, or 4 μM of pravastatin (Cayman, Ann Arbor, MI, USA), and stimulated with 30 μg/ml cholesterol (Sigma, St Louis, MO, USA) plus 1 μg/ml 25-hydroxycholesterol (Sigma). HK-2 and HepG2 cells were treated with 1, 2, or 4 μM pravastatin plus 25-hydroxy cholesterol and cholesterol for either 24 or 48 h. The expression of pyruvate kinase isozymes L/R (PKLR), PFK-1, PEPCK, and G6PC proteins was then examined by western blotting.

### Animals

#### High-fat-diet-fed mice experiment

Four-week-old male C57BL/6J mice were housed under controlled conditions (21 °C ± 2 °C, 60% ± 10% humidity, 12-h light/12-h dark cycle) with *ad libitum* access to food and water. After 1week, the mice were divided into 2 groups according to treatment (untreated control, n = 5; pravastatin, n = 9). Beginning at 5 weeks of age, all mice were fed a high-fat diet that included 45% lipids (Research Diets, Inc., D12451) with or without pravastatin (0.01%, w/w) for 16 weeks.

#### Non high-fat-diet-fed mice experiment

For experiment of pravastatin only treatment, 7 week-old mice were divided into 4 groups comprising 5 mice each (control vs. pravastatin treatment for 2 weeks, and control vs. pravastatin treatment for 4 weeks). After 1 week, the diet for each treatment group was supplemented with 0.01% (w/w) pravastatin. Food intake and body weight were evaluated twice a week at the same time of day. The mice were then anesthetized with 50 mg/kg Zoletil (Zolazepam; Virbac SA, France). Blood samples were then collected by cardiac puncture.

### Immunoblots

Protein extracts from kidney and liver tissue were isolated using a radioimmunoprecipitation assay (RIPA) buffer containing 50 mM Tris-HCl (pH 7.5), 150 mM NaCl, 1% Nonidet P-40, 0.5% sodium deoxycholic acid, and 0.1% SDS. Proteins were boiled for 5 min, separated by 10–15% SDS-PAGE, and blotted onto polyvinylidene difluoride membranes. Proteins expression was detected using the following primary antibodies: PKLR, PFK-1, G6PC, PEPCK (1:1,000) (Cell Signaling Technology, Beverly, MA, USA), and β-actin (1:10,000) (Sigma, USA). Protein bands were detected using an Immobilon Western Chemiluminescent HRP substrate kit (Millipore Corporation, Billerica, MA, USA). The image analysis program ImageJ (NIH; Bethesda, MD) was used for band intensity analysis. Protein concentrations were determined using the bicinchoninic acid (BCA) assay (Sigma-Aldrich).

### Pyruvate kinase (PK) assay

Mouse kidney and liver tissue lysed with PK assay buffer, collected, and PK activity was determined using a PK activity assay kit (ab83432, Abcam, UK) according to the manufacturer’s instructions. In the PKLR activity assay, PEP and ADP are catalyzed by PKLR to generate pyruvate and ATP. One unit of pyruvate kinase is the amount of enzyme that transfers a phosphate group from PEP to ADP, yielding 1.0 μmol of pyruvate/min at 25 °C.

### Blood glucose measurement

Blood glucose (mg/dL) was determined using a portable glucometer (Medisense Companion 2 meter, Medisense Inc., Waltham, MA, USA) according to the manufacturer’s instructions.

### Statistical analysis

Statistical tests were carried out using PRISM (GraphPad Software, San Diego, CA, USA). A value of *P* < 0.05 was considered statistically significant. Comparisons of three or more groups were analyzed by one-way ANOVA (analysis of variance) and post Dunnett’s multiple comparison tests. Data are expressed as mean ± SEM of independent experiments.

## Supplementary information

Supplementary Info
