# Supplementary material for: Reduced expression of pyruvate kinase in kidney proximal tubule cells is a potential mechanism of pravastatin altered glucose metabolism
Source: Sci Rep. 2019 Mar 29;9:5318. doi: 10.1038/s41598-019-39461-2 (PMC6440950; doi:10.1038/s41598-019-39461-2)

**Reduced expression of pyruvate kinase in kidney proximal tubule cells is a potential mechanism of pravastatin altered glucose metabolism**

Yong Pyo Lee^1^†, Yuri Cho^2^†, Eun Jee Kim^2,3^, Hyojung Lee^2^, Hoon Young Choi^4^, Hye Jin Wang^5^, Eun Seok Kang^5^, Yu Seun Kim^2,6^, Myoung Soo Kim^1,2,6^* and Beom Seok Kim^2,7^*

^1^Department of Medicine, The Graduate School, Yonsei University, Seoul, Republic of Korea

^2^The Research Institute for Transplantation, Yonsei University College of Medicine, Seoul, Republic of Korea

^3^Brain Korea 21 PLUS Project for Medical Science, Yonsei University, Seoul, Republic of Korea

^4^Department of Internal Medicine, Yonsei University College of Medicine, Republic of Korea

^5^Division of Endocrinology and Metabolism, Department of Internal Medicine, Yonsei University College of Medicine, Seoul, Republic of Korea

^6^Department of Transplantation Surgery, Severance Hospital, Yonsei University Health System, Seoul, Republic of Korea

^7^Division of Nephrology, Department of Internal Medicine, Severance Hospital, Yonsei University Health System, Seoul, Republic of Korea

†These authors contributed equally to this study.

**Corresponding Author:**

Myoung Soo Kim, M.D., Ph.D.; Department of Surgery, Yonsei University College of Medicine, 50-1 Yonsei-ro, Seodaemun-gu, Seoul 03722, Republic of Korea; Phone: +82-2-2228-2123; E-mail: [ysms91@yuhs.ac](mailto:ysms91@yuhs.ac)

Beom Seok Kim, M.D., Ph.D.; Department of Internal Medicine, Yonsei University College of Medicine 50-1 Yonsei-ro, Seodaemun-gu, Seoul 03722, Republic of Korea; Phone: +82-2-2228-1969; E-mail: [docbsk@yuhs.ac](mailto:docbsk@yuhs.ac)

**E-mail addresses of authors:**

Yong Pyo Lee: [yp_lee@hanmail.net](mailto:yp_lee@hanmail.net), Yuri Cho: [yrcho@yuhs.ac](mailto:yrcho@yuhs.ac), Eun Jee Kim: [eunjk@yuhs.ac](file:///D:\yrcho\desktop\논문%20작업\statin\Sci%20Rep\eunjk@yuhs.ac), Hyojung Lee: [hjlee709@yuhs.ac](mailto:hjlee709@yuhs.ac), HoonYoung Choi: [hychoidr@yuhs.ac](file:///D:\yrcho\desktop\논문%20작업\statin\Sci%20Rep\hychoidr@yuhs.ac), Hye Jin Wang: [gpwlsl00@yuhs.ac](mailto:gpwlsl00@yuhs.ac), Eun Seok Kang: [edgo@yuhs.ac](mailto:edgo@yuhs.ac), Yu Seun Kim: [yukim@yuhs.ac](mailto:yukim@yuhs.ac), Myoung Soo Kim: [ysms91@yuhs.ac](mailto:ysms91@yuhs.ac) and Beom Seok Kim: [docbsk@yuhs.ac](mailto:docbsk@yuhs.ac)

**Supplementary materials and methods**

*Triglyceride and total cholesterol measurement*

Total cholesterol and triglyceride levels were measured with the respective assay kits (ECCH-100 for cholesterol, ETGA-200 for triglyceride) according to the manufacturer’s instructions.

**Supplementary** **Figure 1.** Uncropped images of the western blots presented in Figure1.


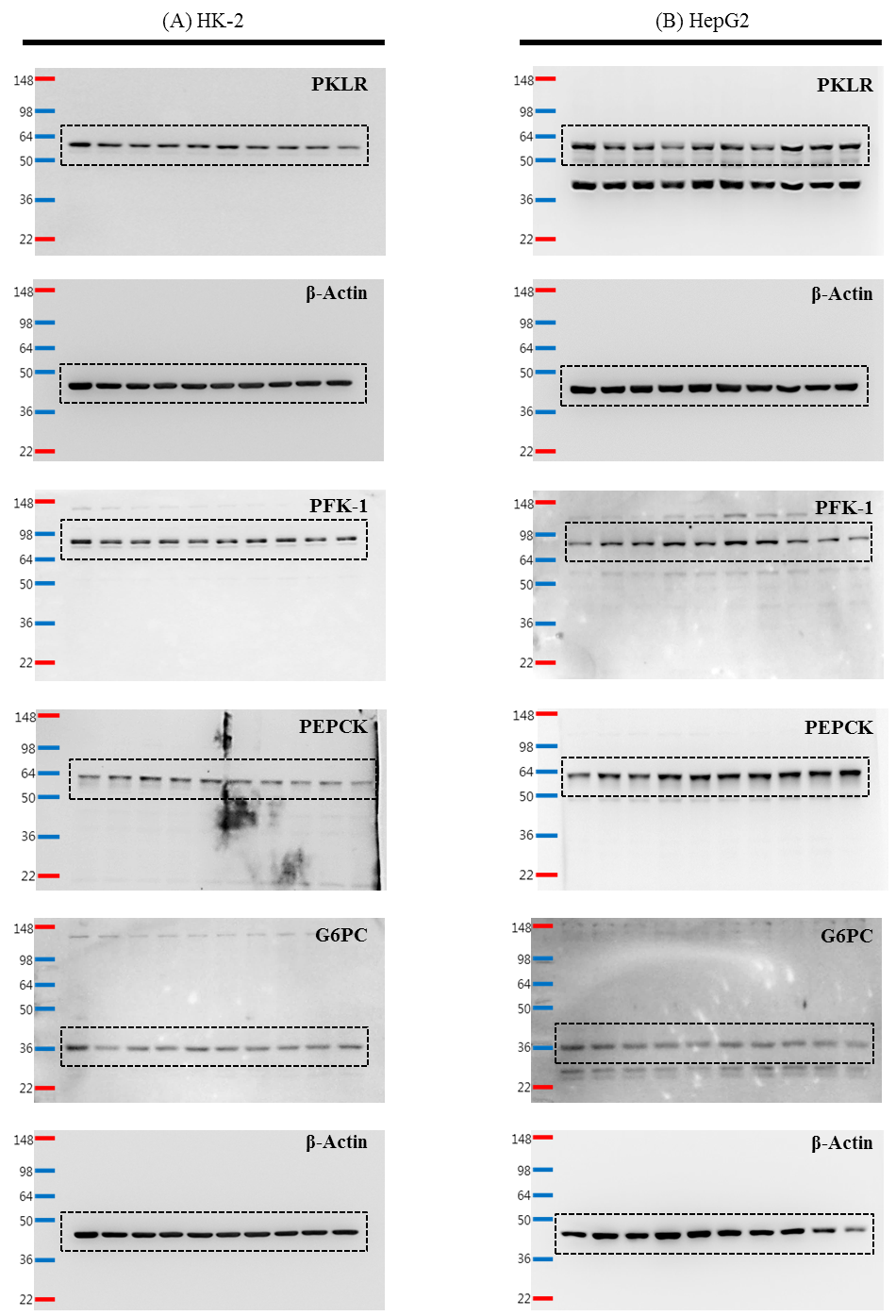


**Supplementary Figure 2.** Quantitative densitometry bar graphs of the blots (PFK-1, PEPCK, and G6PC) in Figure 1


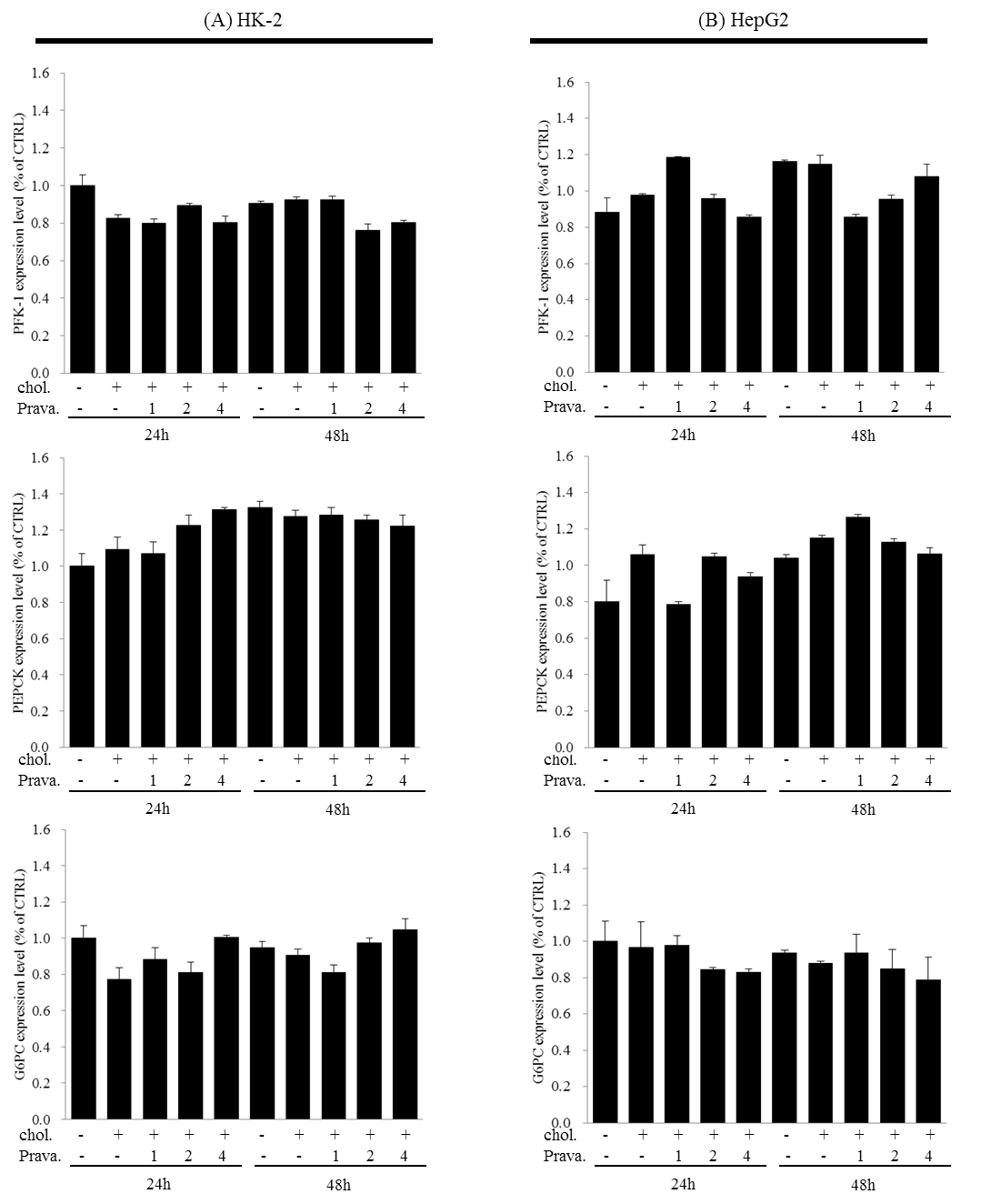


**Supplementary Figure 3.** Uncropped images of the western blots presented in Figure2


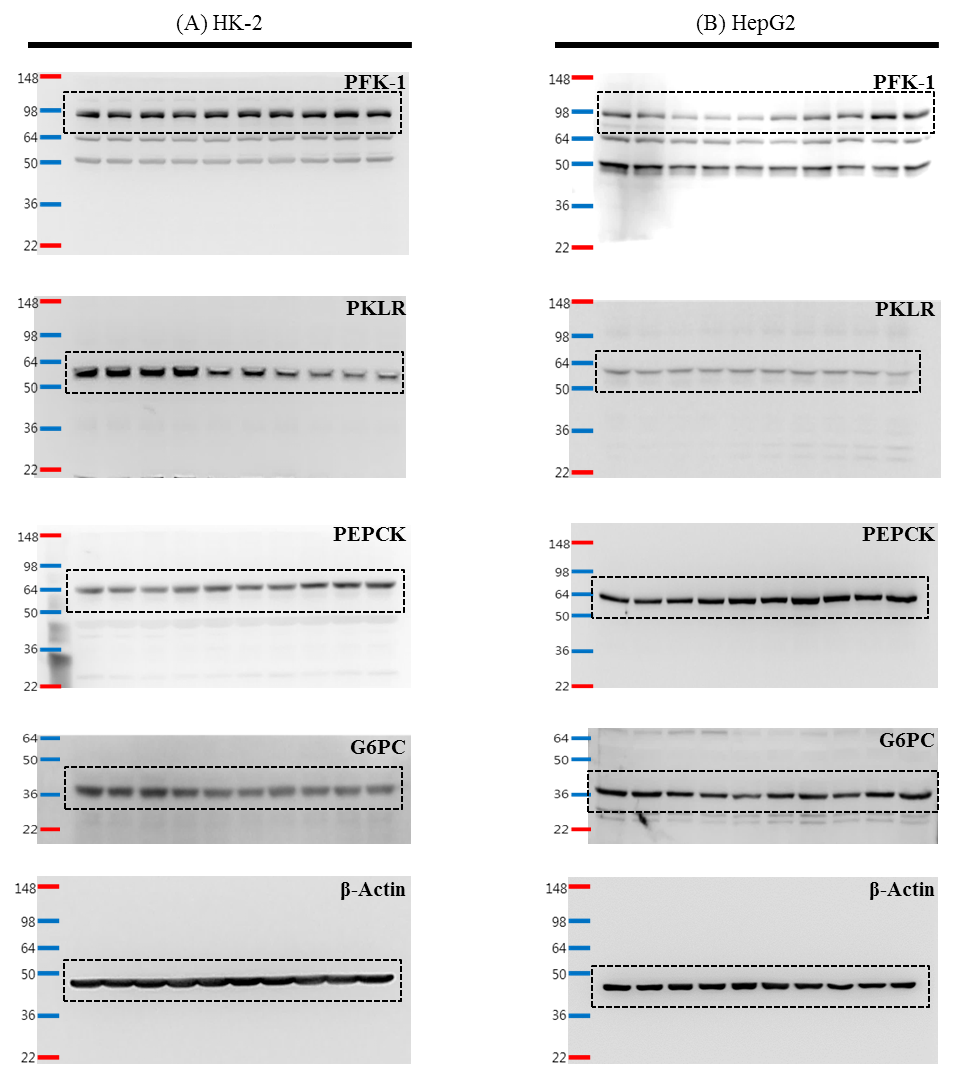


**Supplementary Figure 4.** Quantitative densitometry bar graphs of the blots (PFK-1, PEPCK, and G6PC) in Figure 2


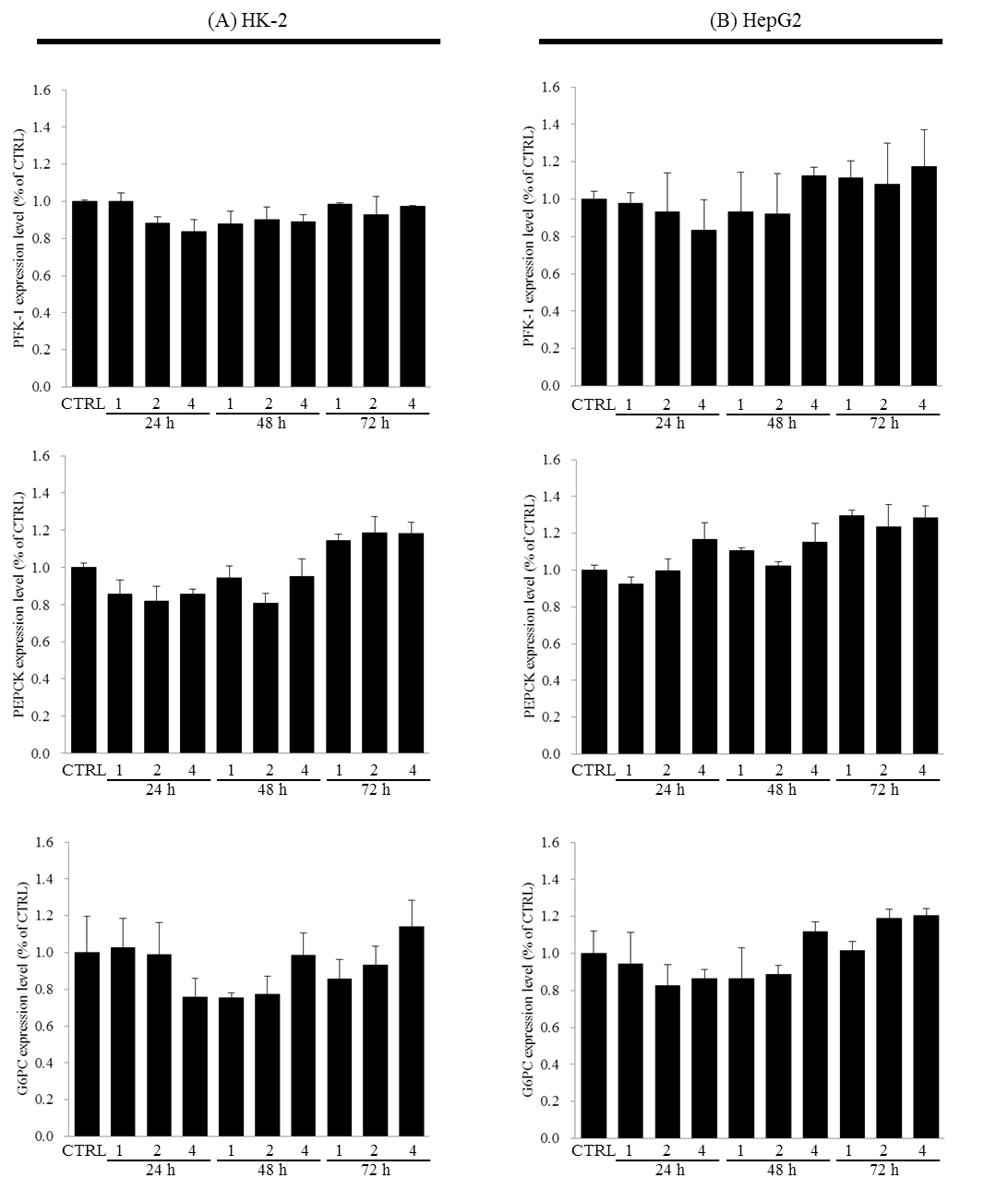


**Supplementary Figure 5.** Uncropped images of the western blots presented in Figure 3


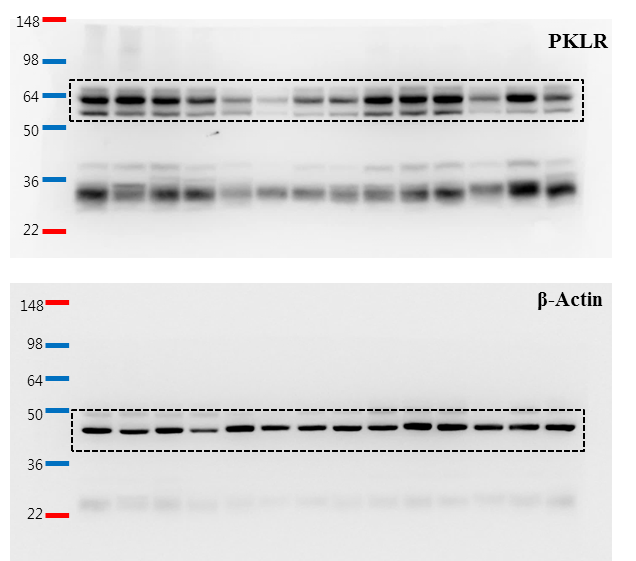


**Supplementary Figure 6.** (A) Total Cholesterol (B) Food intake, and (C) Triglyceride level in high-fat-diet fed mice


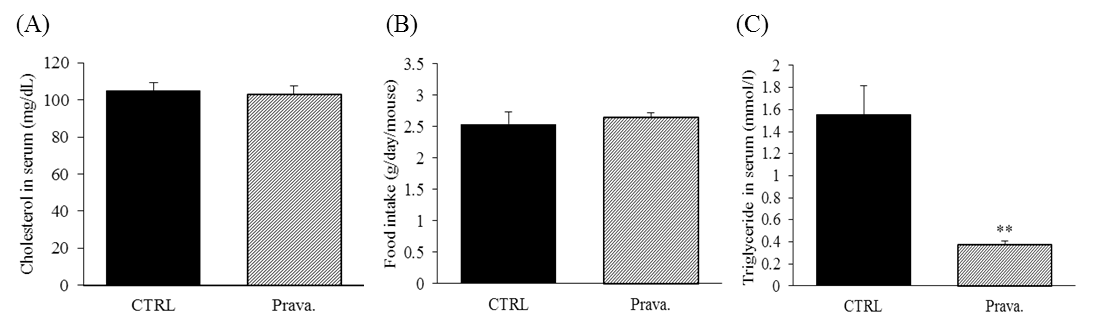


Serum cholesterol (A) and food intake (B) did not differ among the groups. (C) Triglyceride levels in serum were significantly decreased by pravastatin treatments. **, *P* < 0.01 compared with untreated control mice (CTRL, n=9; Prava, n=11).

**Supplementary Figure 7.** Uncropped images of the western blots presented in Figure 4 (A)


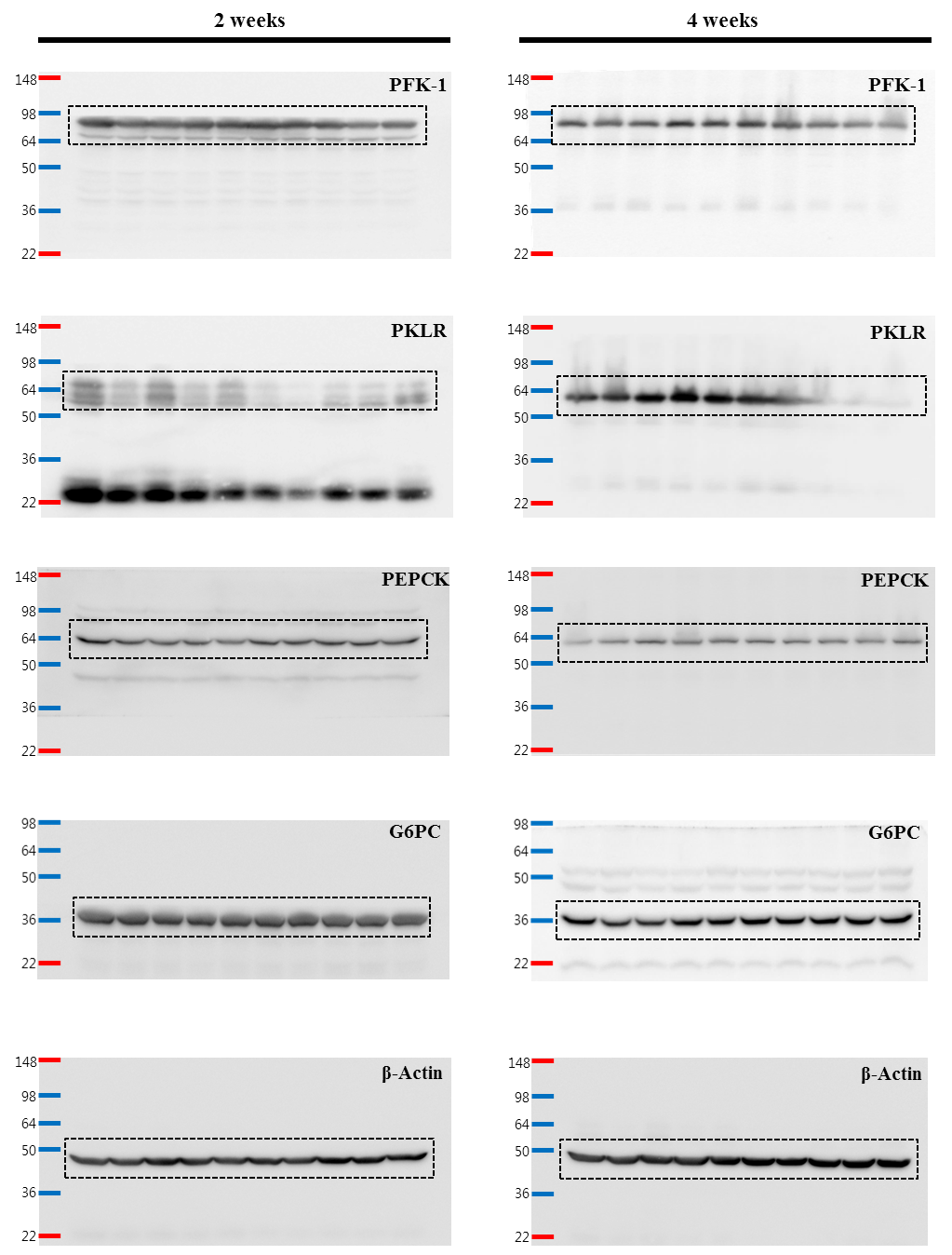


**Supplementary Figure 8.** Uncropped images of the western blots presented in Figure 4 (B)


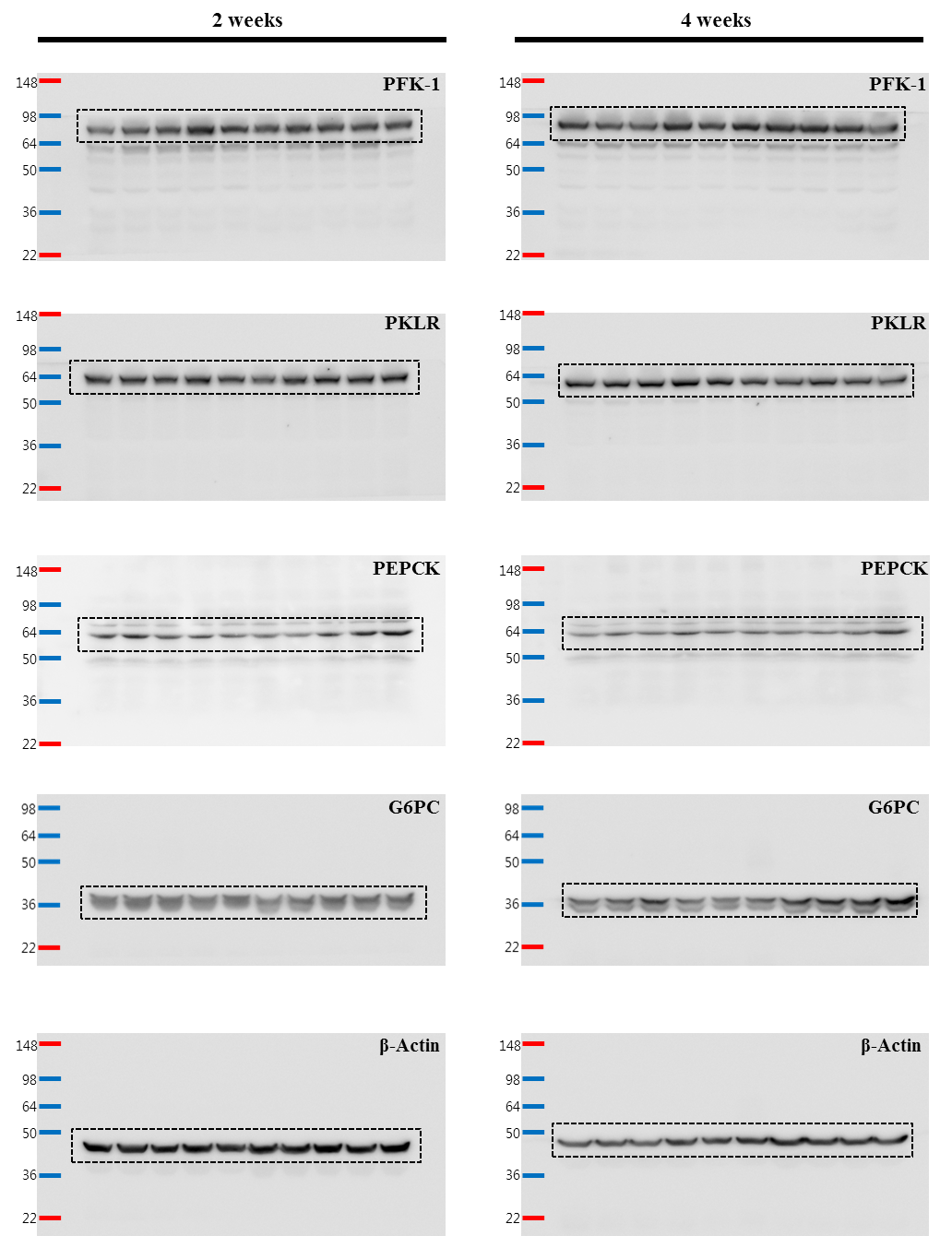


**Supplementary Figure 9.** Quantitative densitometry bar graphs of the blots (PFK-1, PEPCK, and G6PC) in Figure 4


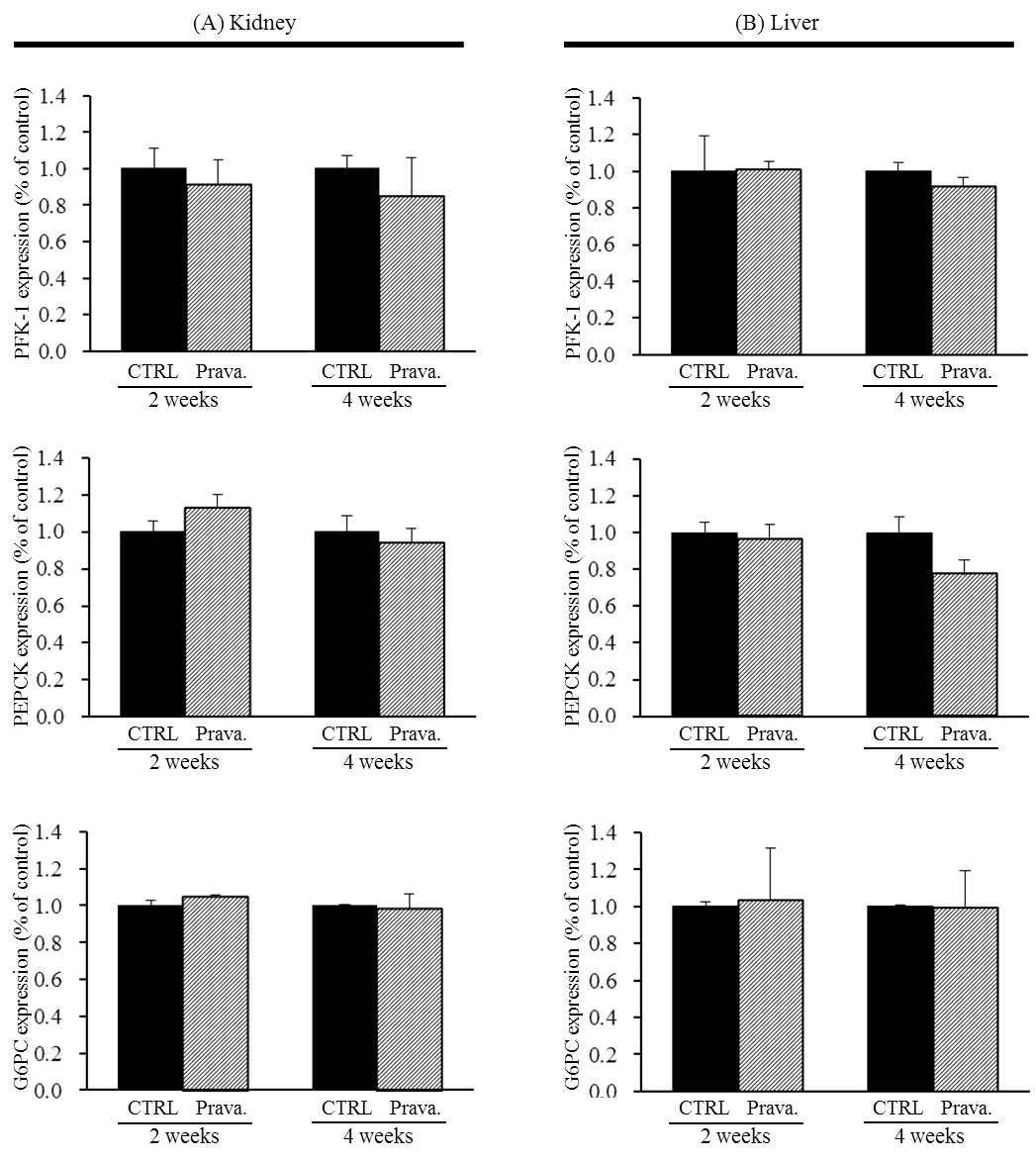

Supplement: Supplementary file 1 — Supplementary Info [file 41598_2019_39461_MOESM1_ESM.docx]
